# A standardized checklist is optimal for patients’ chart check

**DOI:** 10.1002/acm2.12030

**Published:** 2017-01-19

**Authors:** Leonard Kim, Ting Chen, Yi Rong

**Affiliations:** ^1^ Department of Radiation Oncology MD Anderson Cancer Center at Cooper Camden NJ 08103 USA; ^2^ Department of Radiation Oncology Rutgers University New Brunswick NJ 08903 USA; ^3^ Department of Radiation Oncology University of California Davis Comprehensive Cancer Center Sacramento CA 95630 USA

## 1 Introduction

Dr. Leonard Kim is the chief medical physicist at the MD Anderson Cancer Center at Cooper in Camden, New Jersey. Prior to coming to Cooper, he worked at Rutgers University and William Beaumont Hospital. He holds a MS in radiological physics from Wayne State University as well as doctorate and undergraduate degrees from the University of Michigan and the Massachusetts Institute of Technology. Dr. Kim is in support of the proposition.

Dr. Chen obtained his Ph.D. in Bioengineering from University of Pennsylvania in 2003. In the same year he joined New York University as a research scientist conducting research in the field of MRI‐based cardiac imaging and image analysis. He finished his medical physics residency at the Cancer Institute of New Jersey in 2010, and became a faculty member at the same institute after his graduation. His main research interests include medical image analysis, physics modeling in image segmentation and registration, image guided radiation therapy, and new techniques in brachytherapy. He has authored and co‐authored over 40 papers in peer‐reviewed journals. He is currently the lead brachytherapy physicist, and the assistant director of the medical physics residency program at the Radiation Oncology department of Rutgers Cancer Institute of New Jersey. Dr. Chen, on the other hand, has different opinions on this topic.

## 2 Opening statement

### 2.A Dr. Leonard Kim

In 2012, Ford et al. identified “pretreatment plan review by a physicist” as the single most sensitive check against potential incidents.^1^ In 2015, an AAPM practice guideline was released on the development and implementation of safety checklists.^2^ Now with the release of the TG‐100 report,^3^ I argue that, as a logical consequence, a standardized checklist should be used when performing a physicist's check.

The TG‐100 report presents three primary techniques designed to improve quality management: process mapping, failure modes and effects analysis (FMEA), and fault trees. In a fault tree, potential errors in the radiation treatment process (“fault tree inputs”) are organized into a flowchart that shows how these errors can lead to a final, undesirable result such as a treatment error. The individual fault tree inputs can then be linked to a quality control (QC) measure intended to reduce the chance a particular error propagates down the fault tree. Using this framework, we can define the standardized physics checklist simply as the list of all fault tree inputs or process errors for which the physicist's check is the associated QC measure. For example, Figure 1 of TG‐100^3^ shows an example of a fault tree that leads to a patient calculation error. One of the fault tree inputs is “error in calculation algorithm.” If the physicist's check is the associated QC then “calculation algorithm” becomes a standard checklist item. In our clinic, we have broken down this specific item further so that our standard checklist includes checking calculation algorithm, CT density table, heterogeneity correction method, dose grid size and resolution, density overrides, and CT field‐of‐view/patient cutoff, all of which could lead to a dose calculation error by the treatment planning system.

I have suggested a standardized checklist is the list of all potential process errors for which the physicist's check is the designated QC. If a physicist does not use a checklist, he or she is essentially relying on their memorization of the fault tree. I respect the talents and capabilities of medical physicists, but the physics checklist used in our clinic approaches 100 items, each associated with a potential failure, and it is hard to be confident that even a medical physicist can mentally run through a checklist of that size completely and reliably. Using the FMEA concept of “risk priority number” (RPN), a “metric for the risk posed to the patient by undetected failures of the identified type”,^3^ missing a physics check item affects the failure's detectability parameter, increasing the failure's RPN above that intended by the implementation of the physics check in the first place and, by definition, ultimately increasing risk to the patient.

When I argue for a “standardized” checklist, I am suggesting a TG‐100 type of analysis has been performed, and it has been determined which potential failures are to be tied to the physicist's check. In such a framework, there is no ambiguity as to what the checklist items are, and thus the checklist is “standardized.” However, I recognize that checklist organization and usability are important^2^ and that standardizing those characteristics of a checklist may result in varying effectiveness across individual physicists. For this reason, in my clinic, individual physicists are given the freedom to order and organize the standard checklist items to harmonize with their individual checking habits. Each physicist's checklist is made freely available to others so that each can adopt the best organizational and usability features of others.

In conjunction with an active near‐miss reporting system, the efficacy of the checklist is clear in our clinic. The number of near‐miss reports related to items on the standard checklist has dropped, and when they are reported, unfortunately but unsurprisingly, it is often because the checklist was not used. When incident and near‐miss reports suggest we've missed possible failure modes, associated checks are quickly and easily added to the standard checklist. I was once a checklist skeptic, but have been converted. I appreciate the opportunity to argue this position and applaud the steps our profession has taken toward TG‐100's goal: “to apply modern risk‐based analysis techniques to this complex RT process to demonstrate to the RT community that such techniques may help identify more effective and efficient ways to enhance the safety and quality of our treatment processes”.^3^

### 2.B Dr. Ting Chen

Ever since the latest recommendation from TG100,^3^ the use of a standardized checklist in patients’ chart check has become more popular among radiation oncology departments. As an effective tool for error management,^2^ a checklist can, when developed and interpreted appropriately, significantly improve patient safety. However, when implementing it as part of the patient chart QA process, many have misunderstood the inherent scope and limit of a checklist, which may lead to sub‐optimal results.

A standardized checklist, by its nature, was developed to minimize schematic failures.^2^ Little can be achieved, through the use of a checklist, to capture and provide solution to tasks requiring attentional behavior, for example, offline patient setup image review. However, due to the booming development and implementation of new and more sophisticated technologies around the radiation oncology society, more components within the patient chart check workflow are now requiring intellectual judgment from physicists. As a consequence, the overall quality of patients’ chart check has been increasingly relying on “How it was done” instead of “did it or not”. In many scenarios, the way certain QA was performed could determine their final outcomes. For example, for intensity modulated radiotherapy (IMRT), QA devices available to physicists include films, portal dosimtery, 2D phantoms, 3D phantoms, and machine log files; and available analysis tools include film processor, scanner, gamma index‐based software, 3D dose reconstruction software, and MLC leave positioning verification tools. By picking different pairs of QA device and analysis tools, physicists may reach different conclusions based on IMRT QA results even for the same plan delivered through the same radiation platform. Therefore, to evaluate quality and to investigate potential medical errors, technical details of QC should be well documented in addition to a standardized checklist. It should also become common understanding among medical physicists that although a standardized checklist can be useful to avoid unintentional omission of necessary steps in the workflow, which can also be achieved through different means, the overall treatment delivery accuracy is eventually determined by physicists’ understanding on radiation delivery principles and their corresponding appropriate approaches in the chart QC process.

In TG100, and as well as other related reports, the authors were aware of the significant variations between radiation oncology department environments, and avoided giving a universal template checklist for any specific procedures. The reason for this has been clearly stated^2^ that the creation of a checklist, as a human intervention, can be strongly influenced by department's specific infrastructure. Moreover, as has been noted in previous aviation and medical industrial practice, to successfully implement a checklist, continuous efforts are needed to validate, train, evaluate, and improve the checklist. To effectively develop, validate, and maintain the checklist as part of the patient QC, the department should (a) maintain a relatively stable crew and infrastructure; and (b) equipped with resources (both technique and human) to establish an internal or external auditing system to periodically provide objective reviewing opinions about the checklist and its impact to the clinic. Without such auditing scheme, or when there is any major change in a clinical infrastructure, it is possible that a checklist‐based patient chart check may be either defective or incomprehensive in the new environment. Therefore, for small clinics that lack such resources, one should be very cautious in implementing checklist‐based chart check and focus on known evidence‐based best practice.

Although the checklist has been successfully implemented in the aviation industry, we need to notice the difference between professional mission of medical physicists and that of pilots. Pilots’ primary and arguably the most dominate responsibility is safety. It is comparable that by conducting chart check, medical physicists are also responsible for the safety of patients. However, unlike pilots, who in most cases prevent risks by strictly following the safety routine summarized in a standardized checklist, medical physicists are responsible to proactively spot problems within the current system and explore rooms for further improvement. The later requires more creativity to think outside the box. The standardized checklist, on the contrary, enforces the strict obedience of existed scheme. As a medical physicist, it is critical to keep the independent thinking active even when using a standard checklist. This is potentially the best way to get involved in the evaluation and improvement of the checklist‐based patient chart QC process.

In summary, this statement is not suggesting a complete opposition to the use of a standardized checklist in patient chart check, but rather, due to its limitation, checklist‐based patient QC should be implemented under the condition that it is conducted by experienced physicists with thorough understanding of the QC process and techniques within a department with adequate resources. Checklist should not replace the need of proper documentation of procedures and/or forbid independent thinking process of any participants.

## 3 Rebuttal

### 3.A Dr. Leonard Kim

I disagree with the assertion that “checklist‐based patient QA should be implemented under the condition that it was conducted by experienced physicists with thorough understanding of the QA process and techniques within a department with adequate resources.” My contention is that a standardized checklist should be used for patient chart checks regardless of physicist or departmental quality. A particular checklist may not be foolproof or comprehensive, but even an imperfect checklist reduces the frequency of individual risk items not being checked by any physicist. And any such reduction is beneficial, as made explicit by the TG‐100 methodology.

The “Opposed” statement suggests a fear that, if a checklist is implemented, physicists will stop checking items they used to check, if those items are not on the list. Only in this specific scenario, I concede checklist implementation could have a negative effect on departmental quality. But I believe this scenario is unlikely. I do not believe using a checklist discourages physicists from checking something they think should be checked even if it's not on the list. And suppose a physicist with the “creativity to think outside the box” starts checking something that was previously unrecognized as a risk. There is a strong obligation to make sure all of the physicists on the team start checking that item as well. The only way to do that reliably is with a standardized checklist, so that “independent thinking” leads to community benefit.

“He's got ‘em on the list; And they'll none of ‘em be missed”– W.S. Gilbert.

### 3.B Dr. Ting Chen

First I thank Leonard for sharing experience of implementing standard checklist‐based physics QA at his facility. It was great (but not totally surprising) to find out by adopting quality management techniques, such as fault tree, a standardized checklist can be generated and implemented successfully in a complex clinical environment. What impressed me even more was that by allowing physicists to create and share their individual version of checklist, independent thinking among physicists was maintained in the QC process.

However, I would like to use the opportunity to re‐empathize that the standardized checklist should be generated following a scientific approach, the efficiency and effectiveness of the checklist should be continuously monitored for further improvement, and the success of a checklist‐based physics QA system is heavily relying on physicists’ continuous involvements.

In the opening remark, Leonard showed us an easy example to create the physics checklist to avoid patient dose calculation error. By converting fault tree input into checklist items, one can quickly come up with a list for their corresponding physics checks. However, it should be noted that not all the clinical procedures have a straight forward structure that allows the fault tree to be constructed and then converted into a checklist with minimal effort. For many physics associated procedures, a scientifically robust quality management/analysis should be conducted to help the creation of the checklist. Such analysis will avoid missing necessary checklist items, or overloading physicists with duplicate tasks in the QC process.

The implementation of a checklist‐based physics QC should never be treated as an “once‐for‐all” solution. An up‐to‐date checklist requires continuous efforts to maintain and improve. Evidence showed that QC procedures should be updated periodically to keep up with the latest development of new QA devices, technologies, and standards.^4^, ^5^ These updates should be reflected in the physics checklist accordingly. During the implementation of the checklist, newly found errors and problems should be analyzed for their root causes. If an error was related to defects in the checklist, then a change should be made to the checklist itself in a timely manner to avoid any further incidence. Checklist adjustments are also necessary after a personnel change to accommodate different levels of experience, training, and familiarity to the facility.

A standardized checklist helps medical physicists to perform QC in a more organized way to avoid unintentional schematic errors. However, one should not simply assume a “standardized” checklist as a universal or “once‐for‐all” solution. Checklist should not be used as a replacement of necessary procedures or documentation with respect to each individual patient/plan. With or without a checklist, it is critical for the medical physicist who conducted the QC to be aware of his/her responsibility to patient safety throughout the QC process.

As my final conclusion, I want to thank the authors of the TG100 group, as well as other contributors who introduced and promoted the idea of quality management into the field of medical physics, for providing us a scientific way to control the quality of our QC process.

## Conflict of Interest

All authors have no conflicts of interests to disclose.

## References

[1] Ford EC , Terezakis S , Souranis A , Harris K , Gay H , Mutic S . Quality control quantification (QCQ): a tool to measure the value of quality control checks in radiation oncology. Int J Radiat Oncol Biol Phys. 2012;84:e263–e269. 22682808 10.1016/j.ijrobp.2012.04.036

[2] de Fong los Santos LE , Evans S , Ford EC , et al. Medical physics practice guideline 4.a: development, implementation, use and maintenance of safety checklists. J Appl Clin Med Phys. 2015;16:37–59. 10.1120/jacmp.v16i3.5431 PMC5690123 26103502

[3] Huq MS , Frass BA , Dunscombe PB , et al. The report of Task Group 100 of the AAPM: application of risk analysis methods to radiation therapy quality management. Med Phys. 2016;43:4209–4262. 27370140 10.1118/1.4947547 PMC4985013

[4] Klein EE , Hanley J , Bayouth J , et al. Task Group 142 report: quality assurance of medical accelerators. Med Phys. 2009;36:4197–4212. 19810494 10.1118/1.3190392

[5] McEwen M , DeWerd L , Ibbott G , et al. Addendum to the AAPM's TG‐51 protocol for clinical reference dosimetry of high‐energy photon beams. Med Phys. 2014;41:041501. 24694120 10.1118/1.4866223 PMC5148035

